# Geographic heterogeneity in Black-white infant mortality disparities

**DOI:** 10.3389/fpubh.2022.995585

**Published:** 2022-11-03

**Authors:** Marielle Côté-Gendreau, Katie Donnelly Moran

**Affiliations:** ^1^Office of Population Research, Princeton University, Princeton, NJ, United States; ^2^Department of Sociology, Princeton University, Princeton, NJ, United States

**Keywords:** infant mortality, geography, metropolitan, region, race, maternal education

## Abstract

Despite recent decreases in Black infant mortality, racial disparities persist, motivating continued research into factors related to these inequalities. While the inverse association between education and infant mortality has been documented across races, less is known about its geographic heterogeneity. Using vital statistics from the National Center for Health Statistics, this study considers Black-white disparities in infant mortality for births occurring between 2011 and 2015 across regions and metropolitan status of maternal residence. With logistic regressions, we investigate heterogeneity in maternal educational gradients of infant mortality by geographic residence both within and between races. Beyond confirming the well-known relationship between education and infant mortality, our findings document a slight metropolitan advantage for infants born to white mothers as well as lower returns to education for infants born to Black mothers residing in nonmetropolitan counties. We observe a metropolitan advantage for infants born to Black mothers with at least a bachelor's degree, but a metropolitan disadvantage for infants born to Black mothers with less than a high school degree. The South is driving this divergence, pointing to particular mechanisms limiting returns to education for Southern Black mothers in nonmetropolitan areas. This paper's geographic perspective emphasizes that racial infant health disparities are not uniform across the country and cannot be fully understood through individual and household characteristics.

## 1. Introduction

Infant mortality is a key indicator of population health as it reflects the mother's health, environmental context, and access to socioeconomic resources and healthcare (1). Within countries, infant mortality gives important insight into the health of population subgroups and brings to light long-lasting inequalities (2). Infant mortality in the United States far exceeds infant mortality in peer European countries, with geographic heterogeneity and racial disparities contributing to these higher rates (3, 4). Black-white disparities have persisted over time, and in 2015, the infant mortality rate for Black infants was more than double that of white infants (4). This racial gap is also present between mothers of similar socioeconomic status (5–7), which points to the importance of examining health disparities along with related dimensions such as education, economic circumstances, geography, and nativity status.

The heterogeneity of disparities in infant mortality across the country remain not well-understood. In this paper, we look at geographic patterns in infant mortality in the United States through their intersection with racial and educational inequalities. We bring together socioeconomic status, race, geography, and timing of death, which have been identified by previous papers as essential dimensions for understanding infant mortality in the United States. Using the National Center for Health Statistics' complete linked birth/infant death datasets from 2011 to 2015 and 1998 to 2002, we investigate the role of region and metropolitan status on infant mortality for children born to non-Hispanic white and non-Hispanic Black mothers across categories of educational attainment. Spatial approaches to health research have important implications for public policy through informing appropriate targeting of resources and intervention strategies. Additionally, better understanding the intricacies behind Black-white disparities provides insight into the systemic inequalities that Black communities face and their broad impacts on health more generally.

Below we briefly outline related literature before discussing our data and methods. We then present our findings and conclude with a discussion of our results and their significance in the broader context of racial and geographic disparities in health.

## 2. Literature review

In the infant mortality literature, socioeconomic status is frequently proxied with maternal education. Education is expected to lower the probability of infant mortality through two main pathways. On one hand, women are expected to leverage higher education into improved social and economic conditions, such as better housing, financial stability, quality healthcare, and adequate nutrition. On the other hand, education—through increased knowledge—has an effect on the adoption of individual behaviors that impact health, such as smoking, exercising, assigning importance to nutrition, and seeking early prenatal care (8).

Many have documented group disparities in both infant and adult health and mortality along educational gradients. With data from 2007 to 2010, Fishman et al. (7) found that the Black-white gap in infant mortality cannot be accounted for by differential educational attainment, with college-educated Black mothers experiencing higher infant mortality than white mothers with at most a high school degree. Rather, they identified gestational length as a meaningful factor for explaining the racial gap, suggesting that educational attainment does not reflect comparable life experiences for Black and white mothers. Moreover, Green and Hamilton (6) estimated infant mortality by race/ethnicity and nativity in the United States across educational attainment categories between 1998 and 2002. They observed higher mortality of infants born to Black mothers and lower relative returns to education compared to those born to white mothers. Within each ethnoracial group, they also emphasized the lower levels of infant mortality and flatter educational gradients of immigrant mothers compared to their U.S.-born counterparts, reflecting immigrants' well-documented health advantage (9, 10), part of which gets transferred to their descendants (11–14). Explanations emphasize the positive selection of immigrants with respect to health and protective cultural habits. Yet, neither Green and Hamilton (6) nor Fisherman et al. (7) investigated how returns to education with respect to infant mortality vary across the country.

The important role of geography in infant mortality in the United States has been described in numerous studies. With regard to region, the West and Northeast have historically had lower probabilities of infant mortality than the South and Midwest (3, 6, 15). However, little research has addressed how geographic differences manifest across levels of education. Although Montez and Berkman (16) observed similar gradients in adult mortality across races and census regions and trends in these gradients over time, it is unclear whether these findings would hold in the context of infant mortality and across levels of rurality. In recent years, Rossen et al. (17) reported variations in county-level racial disparities in infant mortality, with the Great Lakes, mid-Atlantic, and parts of Florida having the largest Black-white gap. Additionally, Sparks et al. (18), Yao et al. (19), and Luo and Wilkins (20) emphasized the persistent disadvantage of rural infants in their first year of life across multiple contexts. This disadvantage is linked to lower socioeconomic status and limited access to healthcare services and resources in rural areas. However, after controlling for availability of physicians and neonatal care as well as socioeconomic and other local conditions at the county level, Sparks et al. (18) found that rural counties generally have lower neonatal mortality (mortality within the first 4 weeks) than counties located in large metropolitan areas. Yet, the rural disadvantage persisted with regard to postneonatal mortality (mortality in the remainder of the first year of life).

Consistent with Sparks et al. (18), multiple studies have found that mortality in the neonatal and postneonatal periods is associated with distinct causes and has different associations with maternal and neighborhood socioeconomic characteristics. Notably, neonatal mortality tends to have a weaker association with maternal education than postneonatal mortality (6). Neonatal deaths are most likely to derive from pregnancy- and delivery-related factors—such as congenital malformations, pre-maturity, very low birth weight, and delivery complications (21, 22)—as well as issues related to access to and quality of neonatal care. Low birth weight and short gestational length have been identified as the most important predictors of neonatal mortality: in a study of California-born infants in 1995–1997, the Black-white gap in neonatal mortality was entirely explained by Black mothers' higher rates of low birth weight and pre-term birth (23). Mortality in the postneonatal period, on the other hand, reflects the continuing effects of pregnancy-related complications as well as the role of environment- and household-related factors, with congenital abnormalities, accidents, and sudden infant death syndrome ranking as the leading causes of death (21, 22).

The timing of infant death across neonatal and postneonatal periods has important implications for understanding inequalities in infant mortality. Chen et al. (3) documented that the American disadvantage in infant mortality compared to peer European countries in the period 2000–2005 is driven by postneonatal mortality. Within the United States, regional differences are also primarily explained by differences in postneonatal mortality rather than neonatal mortality. Thus, Chen et al. (3) concluded that the United States' higher infant mortality is due to a steeper socioeconomic gradient as well as large regional differences. These findings speak to the need to better document how the United States' heterogeneity manifests in mortality, and the importance of decomposing infant mortality into its neonatal and postneonatal components.

In light of this review of the literature, this study brings together socioeconomic status, race, geography, and timing of death to further our understanding of the disparities in infant mortality across the United States. In particular, we examine the differences in educational gradients in infant mortality for native-born non-Hispanic Black and white mothers across metropolitan residence and region. First, in line with existing literature, we aim to confirm the negative association between education and infant mortality. Then, the following research questions guide our main analysis:

1. *Heterogeneity within race*. For both Black and white mothers, do returns to education in infant mortality vary across metropolitan status and region?
2. *Heterogeneity in patterns across races*. Do the geographic patterns in returns to education in infant mortality differ between Black and white mothers?

In order to consider the distinct causes of infant mortality throughout the first year of life, we examine the above questions across both the neonatal and postneonatal periods.

Identifying geographic patterns in infant mortality and determining whether Black and white populations share similar patterns is important for two main reasons: first, it informs appropriate allocation of resources; second, it assesses whether geographic heterogeneity contributes to the overall racial gap in infant mortality. We expect significant overlap between our results and the robust body of literature on infant mortality in the U.S., which has documented the persistence of racial disparities and educational gradients. Beyond these well-known characteristics of U.S. infant mortality, this study seeks to shed light on the understudied intersection of race, education, and geography. Our approach of estimating mortality across educational categories is in line with recent research drawing attention to the role of compositional differences in education in time-space comparisons of mortality (24, 25).

## 3. Data and methods

### 3.1. Data

This paper uses data from the National Vital Statistics Birth Cohort Linked Birth/Infant Death Data (LBID) from the National Center for Health Statistics (NCHS) (26). This dataset contains almost all infants born in the United States during a given year linked with information from their death record if death occurred within the first 365 days. Coverage of births is quasi-exhaustive, and we use the restricted data in order to have complete data on mothers' county of residence. We first look at the years 2011–2015, which correspond to the five most recent years for which these data are available. However, the coverage of our data is incomplete due to the adoption of a revised birth certificate form in 2003. While some states immediately switched to the new form, it was not until 2016 that all states were using it. In 2011, 14 states were not using the revised form, and in 2015, two states had still not made the change.

The revised birth certificate form affected the recording of maternal educational attainment and race. The revised birth certificate issued in 2003 reports educational attainment in terms of highest educational level completed, rather than years of schooling which was collected by the 1989 birth certificate format. With regard to race, the revised 2003 birth certificate allows for the reporting of multiple racial identities, whereas the 1989 birth certificate form only allowed one race to be recorded for each parent. With the revised form, the NCHS created a *bridged* race variable transforming multiple race responses into one single race, allowing for continuity with the older records. We use the NCHS bridged race in this analysis. In both time periods, we use NCHS's imputation when racial information was missing from the birth certificate. Additionally, in 2011, NCHS began only releasing maternal education and race data for births that were recorded with the revised birth certificate form. Therefore, in the 2011–2015 time period, we only observe these maternal characteristics for mothers who gave birth in states that had adopted the 2003 revised birth certificate.

Thus, while the LBID has quasi-exhaustive coverage, we are limited to analyzing births that were recorded with the revised form. Because of our incomplete coverage of states in the period 2011–2015 and our focus on geography, we reproduce our analysis in two ways. First, we turn to the years 1998–2002, which are the 5 years that immediately precede the adoption of the revised birth certificate. Although the main purpose of this robustness check is to examine consistency and continuity in geographic patterns, this also allows us to consider long run trends in levels and compare our findings to previous work, notably Green and Hamilton (6). Next, restricting to states that had adopted the revised birth certificate before 2011, we examine whether patterns are stable across the full and restricted set of states in both time periods.

Our main geographic variable of interest is maternal county of residence's metropolitan status. This is a binary variable defined as metropolitan or nonmetropolitan according to whether or not the county was located in a metropolitan statistical area (MSA) as of 2005, i.e., an urban cluster with a population of at least 50,000. More precisely, we use the 2006 NCHS Urban-Rural Classification Scheme for Counties, which places U.S. counties into six categories: large central metropolitan, large fringe metropolitan, medium metropolitan, small metropolitan, micropolitan, and non-core. Large central metropolitan and large fringe metropolitan counties form the core and peripheral counties of MSAs of at least 1,000,000 people. All counties in MSAs with populations in the intervals 250,000–999,999 and 50,000–249,999 are categorized, respectively, as medium and small metropolitan counties. Micropolitan counties belong to urban clusters with population of less than 50,000 and, along with non-core—or rural—counties, form the nonmetropolitan counties in our analysis. We use the 2006 classification, because it is a midpoint between the two time periods under study.

Across our analysis, we restrict the dataset to singletons born to U.S.-born mothers between the ages of 18 and 46 living in the United States at the time of giving birth and who reported a racial/ethnic identity of either non-Hispanic white or non-Hispanic Black^1^ and have non-missing information on maternal education and the covariates of interest. Green and Hamilton (6) show that foreign-born mothers have lower levels of infant mortality and display a weaker association between infant mortality and educational attainment. Interpreting these differences in educational gradients is challenging, because it is unknown from the data how long foreign-born mothers have lived in the U.S. and where they were educated. The inclusion of foreign-born mothers in our analysis posed challenges to the interpretation of our findings. For these reasons, we restrict our analysis to U.S.-born mothers. In the two time periods, we pool births from the 5 years.

### 3.2. Methods

#### 3.2.1. Main analysis

To examine the relationship between infant mortality, education, and metropolitan county residence, we run separate logistic regressions on infant death for infants born to non-Hispanic white and non-Hispanic Black mothers between 2011 and 2015:


(1) $$Logit(mortality_{i}|C_{i},E_{i},X_{i})=\beta_{0}+\beta_{1}C_{i}+\beta_{2}E_{i}+\beta_{3}C_{i}E_{i}+X_{i}^{{}^{\prime }}\alpha$$

where *mortality*_*i*_ is infant mortality, *C*_*i*_ is county of residence's metropolitan status, *E*_*i*_ is mother's educational attainment, *C*_*i*_*E*_*i*_ is the interaction between metropolitan residence and educational attainment, and $X_{i}^{{}^{\prime }}$ is the vector of controls. We follow Green and Hamilton's (6) model specifications and control for mother's age, age squared, marital status, first trimester prenatal care, child's sex, birth order, birth year, and U.S. region of birth (Northeast, Midwest, South, and West). We also produce robust standard errors clustered by U.S. census region to reflect that regional characteristics likely cause observations in the same region to be correlated, and we present 95% confidence intervals.^2^ For each race, we run both a baseline model and a fully specified model. In the baseline model (Model 1), we only include maternal age and maternal age squared in the vector of controls $X_{i}^{{}^{\prime }}$, recognizing that maternal age is a main driver of infant mortality and has a non-linear relationship with infant death (28). In the fully specified model (Model 2), we include the full set of controls as listed above. To consider the different underlying causes of infant mortality, we also conduct the above analysis separately for neonatal and postneonatal mortality where *mortality*_*i*_ is infant death in days 0–27 and in days 28–364, respectively.

Then, to address the question of how the interaction between race and education varies over geography, we conduct this analysis broken out by U.S. region. The model specification is as follows:


(2) $$\begin{array}{l} Logit(mortality_{i}|C_{i},R_{i},E_{i},X_{i})=\beta_{0}+\beta_{1}C_{i}+\beta_{2}R_{i}+\beta_{3}E_{i}\\ \;+\beta_{4}C_{i}R_{i}+\beta_{5}C_{i}E_{i}+\beta_{6}R_{i}E_{i}\\ \;+\beta_{7}C_{i}R_{i}E_{i}+X_{i}^{{}^{\prime }}\alpha \end{array}$$

where variables are as defined above with the inclusion of the complete interaction between county of residence's metropolitan status, maternal educational attainment, and U.S. region of birth (Northeast, Midwest, South, and West).

In figures, we present the total predicted probabilities of infant, neonatal, and postneonatal mortality. These predicted probabilities are computed by averaging over predicted probabilities for each mother in the dataset, using the regression coefficients. Thus, the estimates shown in the figures below do not eliminate differences in distributions of covariates between subgroups and, rather, represent the average predicted probability of infant mortality for mothers in these subgroups.

When logistic regression is used to model rare events, there are potential concerns related to the low number of events observed in the data (29). Past research has suggested that thresholds as low as 10 (30) or even 5 (31) events per variable included in the model produce valid estimates. Because our coverage of infant mortality in the LBID is quasi-exhaustive, we observe 7, 080 instances of mortality (323 per predictor) in our most restrictive model with the largest number of coefficients (Black mothers, postneonatal mortality, Equation 1, Model 2). The bias in logistic regression models fitted by maximum likelihood has been found to be minimal with much smaller sample sizes and fewer events per variable than in the LBID (32–36). Therefore, while infant mortality is a rare event, it is not rare enough in our data to bias our logistic regression estimates.

#### 3.2.2. Supplemental analyses

We conduct two supplemental analyses. First, to alleviate data quality concerns with the incomplete coverage of births in the 2011–2015 data and consider long run trends, we repeat the above analysis for births that occurred between 1998 and 2002. These are the 5 years that directly preceded the adoption of the revised birth certificate and are thus the most recent years for which data are complete with respect to our variables of interest. Moreover, this allows for direct comparison with Green and Hamilton (6), who used the 1998–2002 period for the same reasons. Second, we repeat the main analysis on both time periods (2011–2015 and 1998–2002) restricting to the subset of states that had switched to the 2003 revised birth certificate form by 2011 in order to examine the possibility that the missing and non-missing states are fundamentally different and restricting the analysis to a subset of states is driving some of the results.

## 4. Findings

### 4.1. Description of the sample

Our main analysis is conducted on 10, 343, 382 births which occurred between 2011 and 2015. Before restricting our sample, we have record of 19, 849, 690 infant births. Restricting to singletons born to U.S.-born non-Hispanic white or non-Hispanic Black mothers between the ages of 18 and 46 residing in the U.S. at the time of birth brings this figure down to 11, 786, 983. Before making this restriction, mother's country of birth and race were missing from 0.3% and 4.3% of records, respectively. We keep records with imputed maternal race; 18.85% of our final sample have an imputed value for race. In terms of our main independent variables of interest, we observe maternal county of residence—and thus metropolitan status—as well as U.S. region of birth for all births to mothers residing in the U.S. Due to the changes in the birth certificate format discussed above, we do not observe maternal education for over 1 million (8.67%) records. While unobserved maternal education is not correlated with metropolitan county status, it is not evenly distributed geographically: whereas 20.87% births in the Northeast are missing maternal education, almost all education is observed in the Midwest (99.36%). Of our covariates—maternal age, marital status, prenatal care, child's sex, birth order, birth year, and U.S. region of birth—we are only missing values for birth order (0.54%) and prenatal care (11.53%). Restricting to observations for which we observe maternal education and have non-missing covariates brings us to the final analytic sample of 10, 343, 382 births.

Table 1 summarizes our key variables by race and maternal metropolitan status. Of the births that we analyze, 81% are to white mothers. A larger proportion of white mothers reside in nonmetropolitan counties than Black mothers—22% and 10%, respectively. Infant mortality—at any point in the first year—occurs more among infants born to Black mothers. white mothers living in metropolitan counties have the largest proportion with at least a bachelor's degree, whereas only 7% of Black mothers living in the nonmetropolitan South have at least a bachelor's degree. While a majority of metropolitan Black mothers live in the South (55%), 89% of nonmetropolitan Black mothers live in the South. white mothers in metropolitan counties are on average older than white mothers in nonmetropolitan counties and Black mothers regardless of county of residence. Fewer Black mothers are married than white mothers. A disproportionate number of births in our analytic sample come from births that occurred in 2014 and 2015 because of states' staggered adoption of the 2003 revised birth certificate form. Table 2 summarizes the same variables for the sample of 12, 303, 635 births that occurred between 1998 and 2002. The 1998–2002 data are used to consider long run trends in infant mortality and the robustness of our results due to the unobserved births in 2011–2015 in states that had not yet adopted the revised form.

**Table 1:** Summary statistics by race and metropolitan county of residence for births between 2011 and 2015.

	**All**	**White**		**Black**	
		**Metro**	**Nonmetro**	**Metro**	**Nonmetro**
Infant mortality (per 1,000 births)					
Infant mortality	5.10	3.80	5.06	9.46	9.80
Neonatal mortality	3.08	2.29	2.95	5.86	5.92
Postneonatal mortality	2.02	1.52	2.12	3.62	3.90
Maternal educational attainment					
< High school	0.09	0.06	0.12	0.15	0.17
High school	0.25	0.20	0.30	0.34	0.41
Some college (no degree, associate)	0.33	0.31	0.36	0.37	0.35
Bachelor's degree +	0.33	0.42	0.22	0.14	0.07
Region of birth					
Northeast	0.14	0.15	0.11	0.11	0.02
Midwest	0.29	0.29	0.40	0.24	0.08
South	0.40	0.35	0.37	0.55	0.89
West	0.17	0.21	0.13	0.09	0.01
Infant characteristics					
Male	0.51	0.51	0.51	0.51	0.51
Maternal characteristics					
Age (mean)	28.12	29.00	26.99	26.34	25.29
(SD)	(5.58)	(5.43)	(5.35)	(5.65)	(5.23)
Married	0.61	0.72	0.64	0.24	0.20
Prenatal care in the 1st trimester	0.78	0.82	0.77	0.66	0.65
No previous births	0.40	0.42	0.38	0.37	0.35
1 previous birth	0.33	0.33	0.33	0.29	0.30
2 previous births	0.16	0.15	0.18	0.18	0.19
3+ previous births	0.11	0.09	0.12	0.16	0.16
Year of birth					
2011	0.19	0.19	0.18	0.18	0.16
2012	0.19	0.19	0.19	0.19	0.16
2013	0.20	0.20	0.20	0.20	0.21
2014	0.21	0.21	0.22	0.21	0.24
2015	0.22	0.21	0.22	0.22	0.24
***n***	10,343,382	6,556,951	1,837,069	1,747,672	201,690

Values reported are proportions unless otherwise noted.

**Table 2:** Summary statistics by race/ethnicity and metropolitan county residence for births between 1998 and 2002.

	**All**	**White**		**Black**	
		**Metro**	**Nonmetro**	**Metro**	**Nonmetro**
Infant mortality (per 1,000 births)					
Infant mortality	5.83	4.33	5.54	11.49	11.98
Neonatal mortality	3.61	2.69	3.25	7.27	7.69
Postneonatal mortality	2.23	1.65	2.30	4.25	4.32
Maternal educational attainment					
< 12 years	0.12	0.09	0.15	0.20	0.23
12 years	0.34	0.30	0.40	0.42	0.50
13–15 years	0.25	0.25	0.26	0.26	0.21
≥16 years	0.29	0.36	0.19	0.12	0.06
Region of birth					
Northeast	0.17	0.19	0.11	0.14	0.01
Midwest	0.27	0.26	0.37	0.23	0.05
South	0.39	0.34	0.40	0.53	0.93
West	0.17	0.20	0.12	0.09	0.01
Infant characteristics					
Male	0.51	0.51	0.51	0.51	0.51
Maternal characteristics					
Age (mean)	27.66	28.58	26.45	25.62	24.43
(SD)	(5.86)	(5.76)	(5.57)	(5.77)	(5.36)
Married	0.70	0.80	0.75	0.31	0.27
Prenatal care in the 1st trimester	0.87	0.90	0.86	0.76	0.73
No previous births	0.39	0.41	0.38	0.34	0.33
1 previous birth	0.34	0.35	0.35	0.31	0.33
2 previous births	0.17	0.16	0.17	0.19	0.20
3+ previous births	0.10	0.08	0.10	0.16	0.14
Year of birth					
1998	0.20	0.20	0.20	0.20	0.20
1999	0.20	0.20	0.20	0.20	0.20
2000	0.20	0.20	0.20	0.20	0.21
2001	0.20	0.20	0.20	0.20	0.20
2002	0.20	0.20	0.20	0.20	0.19
***n***	12,303,635	8,004,620	2,089,123	1,937,565	272,327

Values reported are proportions unless otherwise noted.

### 4.2. Confirmation of educational gradients in infant mortality

**Figure 1:**
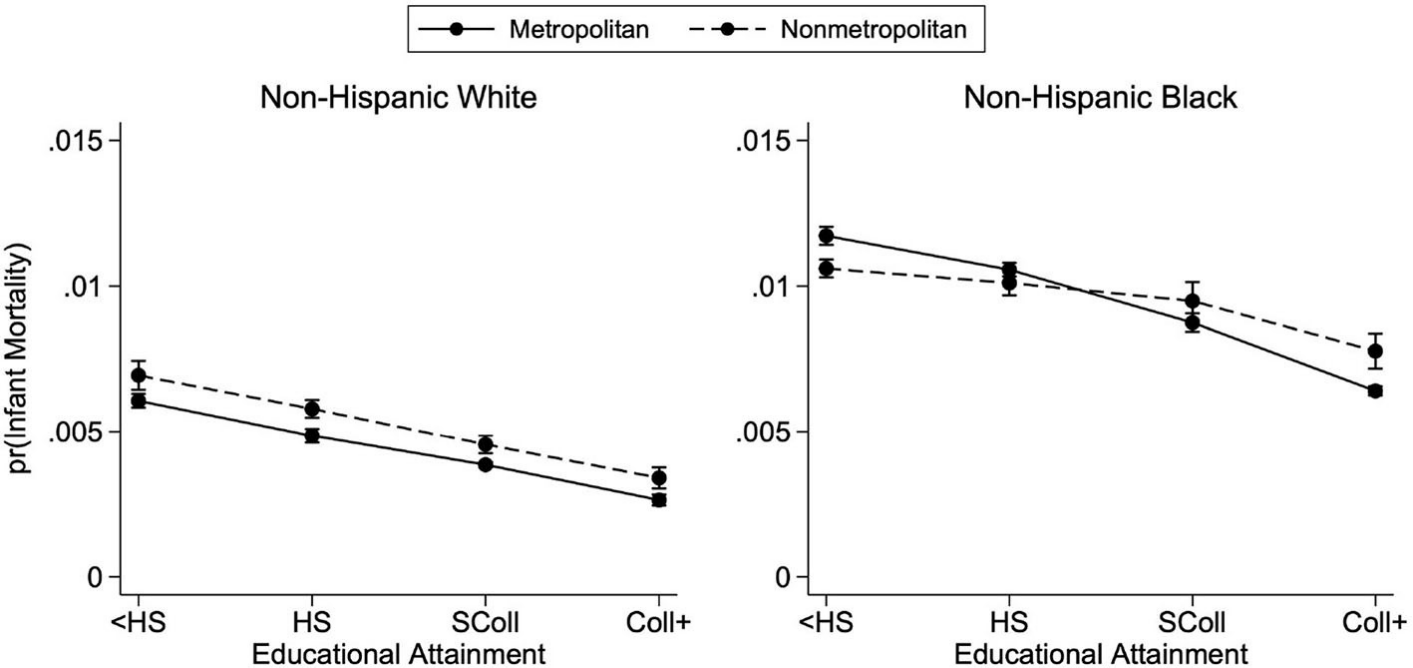
Predicted probabilities of infant mortality by metropolitan residence and race, 2011–2015. Predicted probabilities are from logistic regressions controlling for mother's age, mother's marital status, first trimester prenatal care, child's sex, birth order, birth year, U.S. region of birth, and mother's county of residence's metropolitan status. See Table 3 for full regression results. Births occurred between the years 2011 and 2015. Educational attainment is observed only from the revised birth certificates which had not yet been adopted by every state.

Figure 1 presents the educational gradients of infant mortality by metropolitan status of county of residence for births occurring between 2011 and 2015, separately for white and Black mothers. These predicted probabilities of infant mortality—and all predicted probabilities presented in our figures—are calculated from the regression coefficients of the fully specified model (Table 3, Model 2). From the regression results in Table 3, maternal age and education are negatively associated with the probability of infant mortality in the baseline model (Model 1). However, the effect of age is eliminated after controlling for additional maternal and infant characteristics in the fully specified model (Model 2). In Model 2, for both white and Black mothers, being married and starting prenatal care in the first trimester are negatively associated with the probability of infant mortality. In all models, region is significantly associated with infant mortality, with the Northeast having the lowest probability of infant mortality.

**Table 3:** Logistic regression models of infant mortality stratified by race, 2011–2015.

	**Whites**		**Blacks**	
	**Model 1**	**Model 2**	**Model 1**	**Model 2**
**Maternal demographic**				
**characteristics**				
Nonmetropolitan	0.059	0.069^*^	−0.093^***^	−0.091^***^
	(−0.020, 0.138)	(−0.005, 0.142)	(−0.154, −0.032)	(−0.127, −0.056)
Maternal age	−0.140^***^	−0.106^***^	−0.049^***^	−0.003
	(−0.179, −0.100)	(−0.139, −0.073)	(−0.066, −0.032)	(−0.024, 0.017)
Maternal age^2^	0.002^***^	0.002^***^	0.001^***^	0.000^***^
	(0.002, 0.003)	(0.001, 0.002)	(0.001, 0.001)	(0.000, 0.001)
Mother married		−0.317^***^		−0.099^***^
		(−0.375, −0.259)		(−0.165, −0.034)
**Maternal education**				
High school	−0.325^***^	−0.223^***^	−0.106^***^	−0.106^***^
	(−0.377, −0.272)	(−0.281, −0.166)	(−0.126, −0.086)	(−0.129, −0.083)
Some college	−0.635^***^	−0.457^***^	−0.300^***^	−0.296^***^
	(−0.658, −0.611)	(−0.498, −0.416)	(−0.334, −0.266)	(−0.357, −0.236)
College +	-1.162^***^	−0.837^***^	−0.631^***^	−0.610^***^
	(−1.255, −1.069)	(−0.930, −0.744)	(−0.691, −0.572)	(−0.650, −0.571)
Nonmetro ^*^ Educ				
Nonmetro ^*^ HS	0.060^*^	0.040	0.061^*^	0.057
	(−0.003, 0.124)	(−0.020, 0.100)	(−0.008, 0.129)	(−0.012, 0.127)
Nonmetro ^*^ Some college	0.060^***^	0.032^***^	0.192^***^	0.184^***^
	(0.039, 0.082)	(0.011, 0.052)	(0.066, 0.318)	(0.057, 0.311)
Nonmetro ^*^ College +	0.168^***^	0.118^**^	0.316^***^	0.297^***^
	(0.054, 0.282)	(0.010, 0.226)	(0.217, 0.415)	(0.170, 0.423)
**Child characteristics**				
Child male		0.210^***^		0.179^***^
		(0.178, 0.241)		(0.141, 0.218)
1 prior birth		−0.046^***^		−0.243^***^
		(−0.076, −0.016)		(−0.312, −0.173)
2 prior births		0.107^***^		−0.238^***^
		(0.060, 0.153)		(−0.326, −0.150)
3 or more prior births		0.263^***^		−0.140^***^
		(0.168, 0.357)		(−0.240, −0.040)
1st trimester prenatal care		−0.296^***^		−0.157^***^
		(−0.333, −0.260)		(−0.178, −0.136)
**Region of birth**				
Midwest		0.207^***^		0.254^***^
		(0.197, 0.218)		(0.243, 0.265)
South		0.234^***^		0.170^***^
		(0.228, 0.241)		(0.166, 0.174)
West		0.131^***^		−0.017^***^
		(0.126, 0.135)		(−0.022, −0.012)
**Year of birth**				
2012		−0.010		0.012^*^
		(−0.042, 0.021)		(−0.001, 0.025)
2013		−0.013		−0.027^*^
		(−0.057, 0.031)		(−0.058, 0.004)
2014		0.016		0.013
		(−0.004, 0.035)		(−0.004, 0.030)
2015		0.012		0.039^***^
		(−0.028, 0.052)		(0.015, 0.063)
**Constant**	-2.967^***^	-3.531^***^	-3.958^***^	-4.702^***^
	(−3.521, −2.413)	(−4.044, −3.018)	(−4.152, −3.765)	(−4.978, −4.426)
**Observations**	8,394,020	8,394,020	1,949,362	1,949,362

Robust ci in parentheses.

*** *p* < 0.01,

** *p* < 0.05,

* *p* < 0.1.

The negative association between education and infant mortality is observed in Figure 1: across racial groups, more-educated mothers face a lower probability of infant death than their less-educated counterparts. Additionally, Figure 1 demonstrates the racial disparities in infant mortality. Children born to white mothers with less than a high school education living in nonmetropolitan counties have about the same predicted probability of infant mortality as children born to Black mothers with at least a bachelor's degree living in metropolitan counties. Both of these findings are in line with previous research and are presented here as confirmation of general trends before examining geographic heterogeneity.

### 4.3. Metropolitan gradients

Consistent with previous research, at each level of education, infant mortality tends to be higher in nonmetropolitan counties. Figure 1 demonstrates that the negative association between education and infant mortality is present across metropolitan county status for infants born to both white and Black mothers. However, there is important heterogeneity in this association. For white mothers, the educational gradients are downward sloping for both metropolitan and nonmetropolitan residence, and the probability of infant mortality is consistently higher in nonmetropolitan counties, across all levels of educational attainment; the lines are parallel. For Black mothers, however, this relationship is less consistent. The metropolitan gradient is much steeper than the nonmetropolitan gradient, resulting in nonmetropolitan residence predicting lower probabilities of infant mortality for mothers with less than a high school education, whereas the reverse is true for college-educated mothers.

### 4.4. Regional trends

In this section, we break out the above analysis by region of birth; thus we present educational gradients of infant mortality for white and Black mothers living in metropolitan and nonmetropolitan counties by U.S. region of birth (Northeast, Midwest, South, and West). Before considering these findings, it is important to note that white and Black mothers are not evenly distributed across regions and metropolitan counties (Table 1). The majority of Black mothers live in the South; of all Black mothers who reside in nonmetropolitan counties, 89% live in the South. In contrast, of all white mothers who reside in nonmetropolitan counties, 37% live in the South and 40% live in the Midwest. For both white and Black mothers, a majority live in metropolitan counties. Additionally, it is important to mention that there is geographic variation within the metropolitan and nonmetropolitan categories. Table 4 presents the distribution of births for each of the six NCHS Urban-Rural county classifications by region and race. Across each region (including the South), a higher proportion of Black mothers live in large central metropolitan counties, whereas larger proportions of white mothers live outside of large central metropolitan counties. Thus, while we conduct our analysis along the distinction of metropolitan and nonmetropolitan counties, there is heterogeneity within these categories with white and Black mothers tending to live in different types of metropolitan counties.

**Table 4:** Distribution of births between 2011 and 2015 across 2006 NCHS Urban-Rural Classification Scheme by region and race.

	**Northeast**		**Midwest**		**South**		**West**	
	**White**	**Black**	**White**	**Black**	**White**	**Black**	**White**	**Black**
Metropolitan								
Large central metro	0.20	0.64	0.14	0.53	0.17	0.31	0.33	0.64
Large fringe metro	0.33	0.19	0.24	0.17	0.25	0.21	0.17	0.17
Medium metro	0.24	0.13	0.19	0.18	0.22	0.21	0.23	0.15
Small metro	0.07	0.02	0.14	0.08	0.13	0.11	0.13	0.03
Nonmetropolitan								
Micropolitan	0.12	0.02	0.17	0.03	0.14	0.10	0.10	0.01
Non-core	0.04	0.00	0.12	0.01	0.09	0.05	0.05	0.00
***n***	1,200,707	199,271	2,601,143	439,337	2,953,750	1,144,138	1,638,420	166,616

Values reported are proportions of all births in our analytic sample that were born to mothers living in each of the six county classifications from the 2006 NCHS Urban-Rural Classification Scheme. Proportions are broken out by race and region of birth.

**Figure 2:**
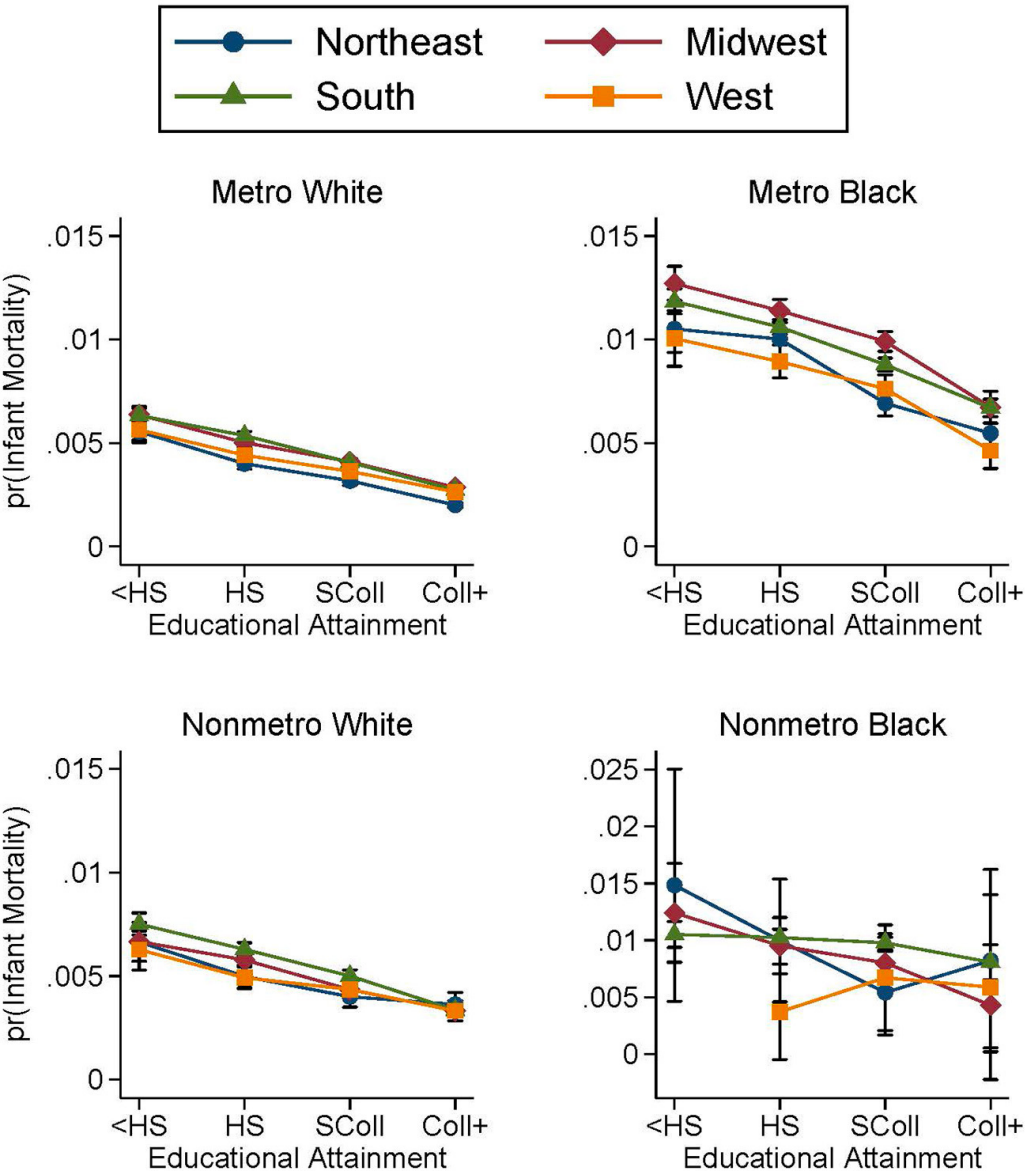
Predicted probabilities of infant mortality by metropolitan residence and region for white and Black mothers, 2011–2015. Predicted probabilities are from logistic regressions controlling for mother's age, mother's marital status, first trimester prenatal care, child's sex, birth order, birth year, U.S. region of birth, and mother's county of residence's metropolitan status as well as the complete interaction between metropolitan status, region, and educational attainment. See Supplementary Table 1 for full regression results. Births occurred between the years 2011 and 2015. Educational attainment is observed only from the revised birth certificates which had not yet been adopted by every state. The top panels plot the educational gradients of infant mortality for white and Black mothers living in metropolitan counties. The bottom panels plot the educational gradients of infant mortality for white and Black mothers living in nonmetropolitan counties.

Figure 2 presents the predicted probabilities of infant mortality, breaking down educational gradients by both metropolitan county status and U.S. region according to the model specifications of Equation 2 (see Supplementary Table 1 for complete regression results). For white mothers, there are only small regional differences in levels and slopes of predicted probability of infant mortality, in both metropolitan and nonmetropolitan counties. Overall, the regional differences are more distinct for Black mothers. In metropolitan counties, the educational gradients of the four U.S. regions have similar slopes and a clear ordering. The Midwest is associated with the highest predicted probability of infant mortality followed by the South. The Northeast and the West have the lowest levels of predicted infant mortality for Black mothers residing in metropolitan counties. The panel for nonmetropolitan Black mothers is noisier, given that sample sizes are very small in the West and the Northeast and that 89% of births occurred in the South. Nevertheless, the remarkable flatness of the nonmetropolitan Southern Black mothers' educational gradient indicates that the flatness observed in Figure 1 is driven by Southern states. This finding does not extend to metropolitan counties. Thus, the South is driving both the nonmetropolitan *advantage* for Black mothers with low education and, on the contrary, the nonmetropolitan *disadvantage* for Black mothers with high education.

### 4.5. Neonatal and postneonatal trends

**Figure 3:**
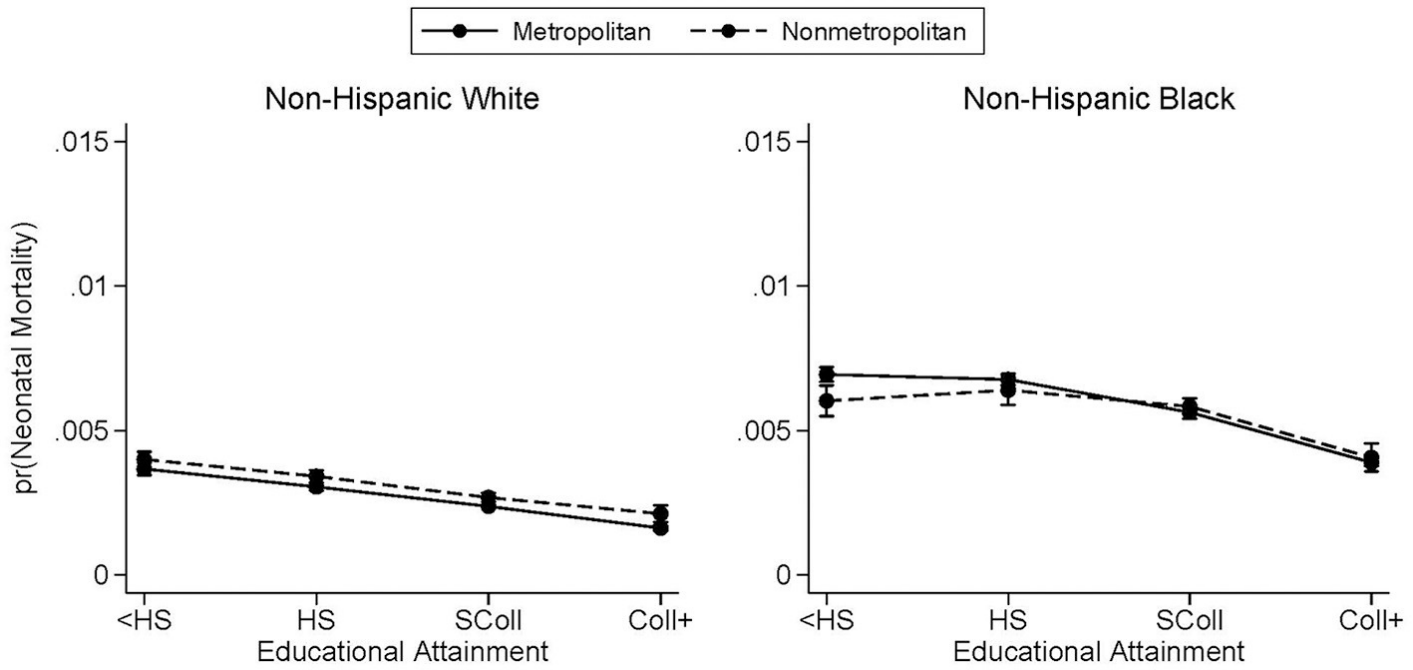
Predicted probabilities of neonatal mortality by metropolitan residence and race. Neonatal mortality is infant death occurring between days 0 and 27. Predicted probabilities are from logistic regressions controlling for mother's age, mother's marital status, first trimester prenatal care, child's sex, birth order, birth year, U.S. region of birth, and mother's county of residence's metropolitan status. See Supplementary Table 2 for full regression results. Births occurred between the years 2011 and 2015. Educational attainment is observed only from the revised birth certificates which had not yet been adopted by every state.

**Figure 4:**
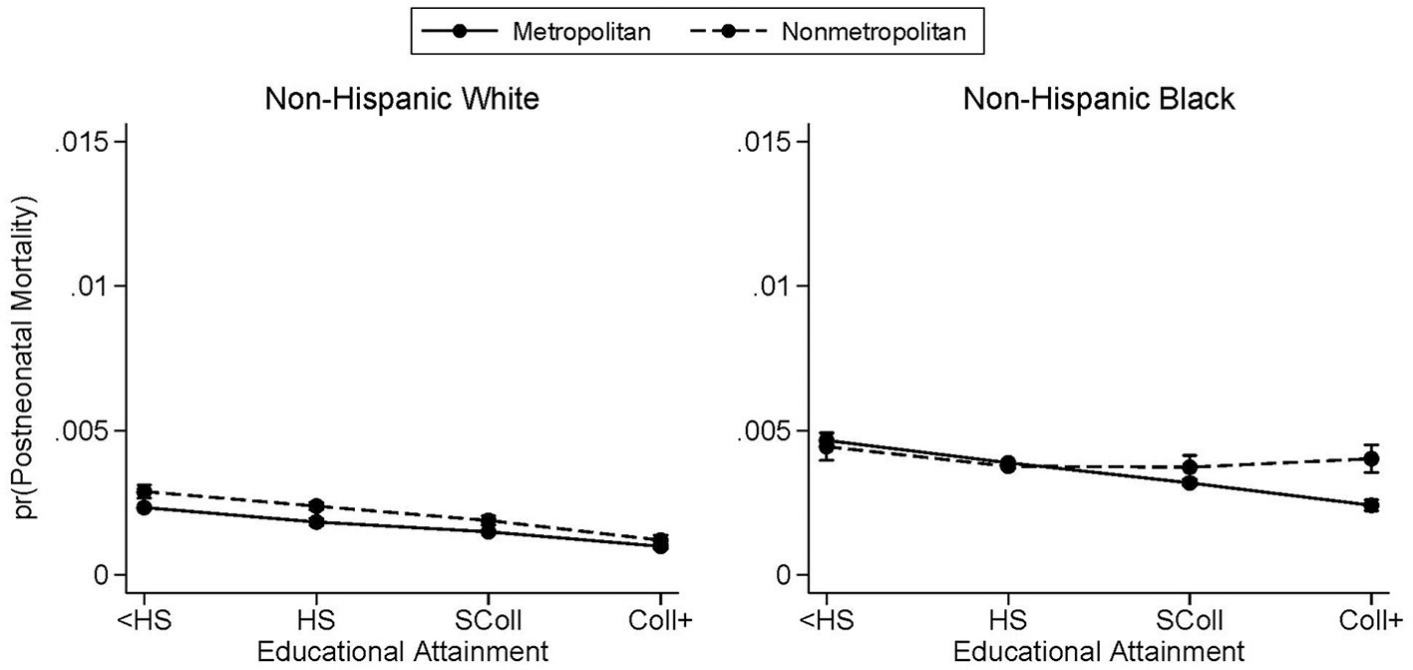
Predicted probabilities of postneonatal mortality by metropolitan residence and race. Postneonatal mortality is infant death occurring between days 28 and 364. Predicted probabilities are from logistic regressions controlling for mother's age, mother's marital status, first trimester prenatal care, child's sex, birth order, birth year, U.S. region of birth, and mother's county of residence's metropolitan status. See Supplementary Table 3 for full regression results. Births occurred between the years 2011 and 2015. Educational attainment is observed only from the revised birth certificates which had not yet been adopted by every state.

The next analysis considers how the educational gradients by metropolitan residence and race vary by timing of infant death. Figures 3, 4 break out Figure 1 by whether the infant died in the first 28 days of life or between days 28 and 364, respectively. For white mothers, the neonatal and postneonatal educational gradients look remarkably similar. In Figures 3, 4, nonmetropolitan county residence is associated with a slightly higher probability of infant mortality—at either time range—for white mothers, and the slopes are very similar between metropolitan and nonmetropolitan counties. There is a greater distinction between the neonatal and postneonatal educational gradients for infants born to Black mothers. Their predicted probabilities of neonatal mortality do not differ between metropolitan and nonmetropolitan counties at any maternal education level besides less than high school (Figure 3). Black mothers with less than a high school education living in metropolitan counties have a higher probability of neonatal mortality than their counterparts residing in nonmetropolitan counties. In terms of postneonatal mortality for infants born to Black mothers, the divergence occurs at the other end of maternal educational attainment (Figure 4). While there is no difference in predicted probability of postneonatal mortality for infants born to Black mothers with lower levels of education, there is a higher probability of postneonatal mortality for infants born to mothers with at least a bachelor's degree and who live in nonmetropolitan counties. Contrary to what was observed in previous studies (6), we find no marked difference in slope between the neonatal and postneonatal gradients.

### 4.6. Robustness checks

#### 4.6.1. Temporal comparison

**Figure 5:**
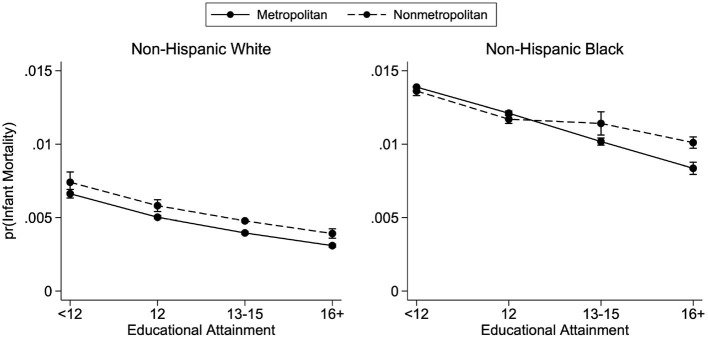
Predicted probabilities of infant mortality by metropolitan residence and race—1998–2002. Predicted probabilities are from logistic regressions controlling for mother's age, mother's marital status, first trimester prenatal care, child's sex, birth order, birth year, U.S. region of birth, and mother's county of residence's metropolitan status. See Supplementary Table 4 for full regression results. Births occurred between the years 1998 and 2002.

Next, we have repeated our analysis looking at educational gradients by metropolitan residence status for an earlier time period where all states could be included. Figure 5 presents the predicted probabilities of infant mortality by race and metropolitan residence status for births that occurred between 1998 and 2002 and suggests largely similar patterns. Again, higher education is associated with lower infant mortality, though there is heterogeneity across race and metropolitan residence status. For infants born between 1998 and 2002 to white mothers, there is a metropolitan advantage with infants born to mothers residing in metropolitan counties having a lower predicted probability of infant mortality at any level of educational attainment. Black mothers' gradients are also negatively sloped and predict higher levels of infant mortality than white mothers. Similar to the 2011–2015 period, the educational gradient is flatter for nonmetropolitan mothers than metropolitan mothers. At the two highest levels of maternal education, there is a higher predicted probability of infant mortality for infants born to Black mothers living in nonmetropolitan counties.

As suggested by recent work, there was a sharp decline in infant mortality for infants born to Black mothers across levels of maternal education and metropolitan residence between 1998–2002 and 2011–2015. Despite this progress, infants born to Black mothers continue to face higher probabilities of infant mortality at any level of education when compared to infants born to white mothers. This time comparison shows a persistence in racial, geographic, and educational patterns in infant mortality, which provides support for our results despite incomplete data. However, it is not possible to more directly compare the two time periods, because, as noted above, the revised birth certificate format changed how maternal education was measured.

#### 4.6.2. Robustness of states included

**Figure 6:**
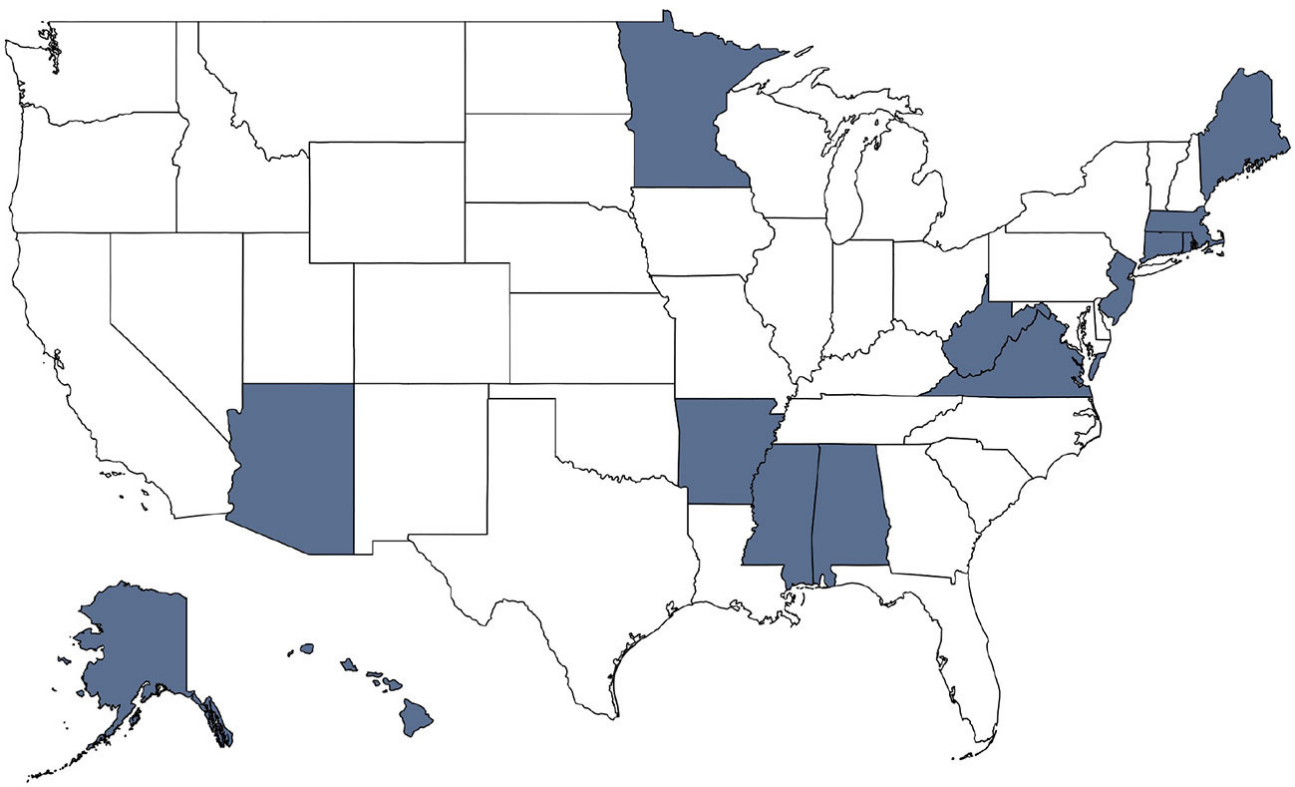
States' adoption of revised birth certificate form by 2011. This map indicates with blue shading the 11 states that had not begun recording births using the 2003 revised birth certificate form before 2011.

**Figure 7:**
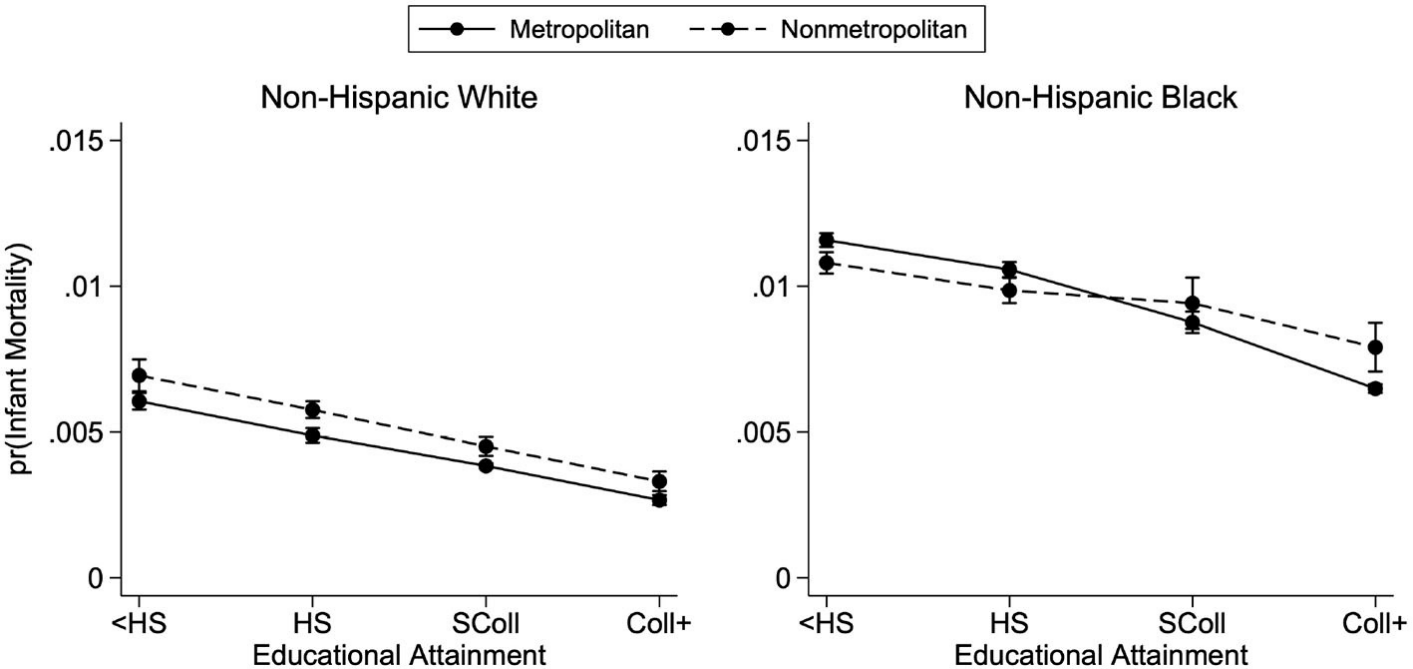
Predicted probabilities of infant mortality by metropolitan residence and race, 2011–2015, state subsample. Educational gradients of infant mortality by race and metropolitan residence for states that had adopted the 2003 revised birth certificate form before 2011. Births occurred between 2011 and 2015. Supplementary Table 5 has full regression results from which the predicted probabilities are calculated. See Figure 1 for complete figure notes and Figure 6 for states included in subsample.

**Figure 8:**
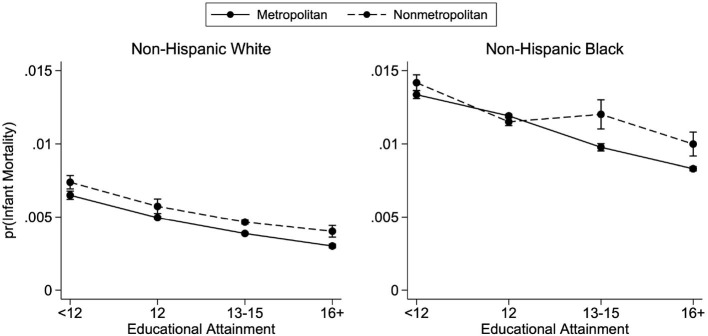
Predicted probabilities of infant mortality by metropolitan residence and race, 1998–2002, state subsample. Educational gradients of infant mortality by race and metropolitan residence for states that had adopted the 2003 revised birth certificate form before 2011. Births occurred between 1998 and 2002. Supplementary Table 6 has full regression results from which the predicted probabilities are calculated. See Figure 5 for complete figure notes and Figure 6 for states included in subsample.

Due to the staggered adoption of the revised birth certificate form, we do not observe maternal education in all states between 2011 and 2015. To address the possibility that the absence of some states in the more recent data is driving some of the changes between the two time periods, we also run the analysis for both time periods restricting the samples to the U.S. states that switched to the revised birth certificate before 2011 (i.e., states for which we have data on maternal education for all years). Figure 6 plots the 14 states that had not yet adopted the revised birth certificate form before 2011. We conduct the above analysis on the 36 states that had revised their form as well as the District of Columbia. Figures 7, 8 plot the educational gradients of infant mortality by race and metropolitan residence for births occurring between 2011–2015 and 1998–2002, respectively, restricting to states that had revised their birth certificates before 2011. We find very similar results in both time periods with this restricted sample. Thus, we conclude that the absent states in the newer years are not driving the observed trends and focus our discussion on the analysis using the full sample of infants for whom we observe maternal education.

## 5. Discussion

Through our findings, we have documented the negative association between maternal education and infant death as well as within-race heterogeneity in the association between education and infant mortality, across both metropolitan status and region. These differences are subtle for infants born to white women but substantial and meaningful for infants born to Black women. The main finding of this paper is the remarkable flatness of the educational gradient of nonmetropolitan Black mothers, driven by Southern states and across both the neonatal and postneonatal periods. This observation could not have been made without this paper's approach looking at the intersection of education and geography. Below we discuss our main findings in more detail for white and Black mothers and some potential mechanisms before outlining this study's limitations and contributions.

We find that geographic differences in infant mortality exist more starkly for infants born to Black mothers than to white mothers. The small regional differences for infants born to white mothers and the consistent, yet small, difference between metropolitan and nonmetropolitan counties suggest that, for white mothers, the detrimental effect of living in a nonmetropolitan county is fairly constant across educational levels at the national scale and for each of the four regions. This finding relates to a broader literature on rural disadvantage that has emphasized difficulties in accessing quality healthcare (37) and also reflects lower maternal age at birth and lower proportions of married mothers, factors that are suggestive of single motherhood as well as higher instability and stress (38).

The relationship between education and geographic residence for infants born to Black mothers, however, requires a more nuanced discussion. Black mothers display distinct patterns of infant mortality when considered through the intersection of both education and geography. Although a marked racial gap exists in all four census regions, the metropolitan Midwest and South contribute the most to the overall Black-white gap in infant mortality. The flat educational gradient observed for nonmetropolitan mothers at the national level is driven by the South, where the vast majority of nonmetropolitan Black mothers live. The difference between metropolitan and nonmetropolitan counties is not constant across levels of maternal education, as evidenced by the crossover between the gradients. At the lowest level of maternal educational attainment, nonmetropolitan mothers fare better than their metropolitan counterparts. This is in contrast to the metropolitan advantage observed for infants born to white mothers as well as the metropolitan advantage that is present for infants born to Black mothers with at least a bachelor's degree.

This geographic pattern has persisted in the past two decades despite the overall decline in infant mortality and the reduction of the absolute gap in infant mortality between Black and white infants, as documented by previous studies (4) and our comparison of the time periods 1998–2002 and 2011–2015. The relative flatness of the educational gradient in infant mortality for nonmetropolitan Black mothers does not explain the overall racial gap. However, it can be interpreted as evidence of low returns to education for infants born to Black mothers in nonmetropolitan Southern counties, which challenges the well-known role of education as a powerful lever for improving health.

These findings point to potentially crucial mechanisms of rural and micropolitan Black health in the United States, with important policy implications. It can be thought that this evidence of low returns to education for the nonmetropolitan Black population in the South, as it manifests through the first year of infancy, stems from more fundamental sources that likely affect a variety of outcomes for which an educational gradient is expected. This finding could be symptomatic of a wider phenomenon impacting multiple facets of life. Below we explore four potential mechanisms that could contribute to this finding. Rather than explanations, these should be taken as avenues for future research.

First, it is possible that the Southern nonmetropolitan counties where these Black mothers live—which includes the Black Belt region known for its high proportions of African Americans and persistent disadvantage through legacies of slavery—offer few opportunities for highly educated mothers to leverage education into higher income and better living situations. Lower observed or perceived quality of education and racial employment discrimination could be a barrier to employment opportunities for college-educated Black mothers. Unfortunately, education is the only measure of socioeconomic status available on U.S. birth certificates, which prevents us from directly testing the hypothesis that the flat educational gradient in infant mortality for nonmetropolitan Black mothers derives from a flatter educational gradient in income.

Second, the selection effect of migration to metropolitan areas could contribute to the Black infant mortality gap between metropolitan and nonmetropolitan areas. Positive selection of migrants with respect to health is generally observed with young internal migrants tending to be healthier than their non-migrant peers. Migration of young adults to metropolitan areas also signals motivation to access better opportunities and living conditions, which could also reflect in higher degrees of health consciousness. However, opportunity-motivated migration comes with potentially detrimental consequences, such as higher stress and weaker social networks (39–41). These mechanisms and their implications for the health gradients of metropolitan and nonmetropolitan populations could be differentiated by race. However, it remains unclear how urban-rural migration within the United States would affect our finding of a flatter educational gradient for Black mothers in nonmetropolitan counties.

Third, given the residential segregation that persists in the U.S. South between Black and white populations, it is possible that nonmetropolitan Black mothers, regardless of education, have worse access to healthcare than their white counterparts, and that health institutions present in predominantly Black counties are of lower quality. The history of racial discrimination in healthcare may also cause Black mothers to receive poorer services or be more reluctant to trust medical professionals even when they do have access to healthcare. For example, Pathman et al. (42) report that Black adults in the rural South experience more dissatisfaction and barriers to care than whites, and research has shown that the racial gap in adverse birth outcomes is connected with levels of racial prejudice in both the county of residence and the county of birth (43). However, access to healthcare cannot account for the entirety of observed trends, in part because it does not explain the steeper educational gradient in metropolitan counties and the nonmetropolitan advantage in neonatal mortality for Black mothers without a high school degree. Landrine and Corral (44) note that residential segregation can have other impacts on health, through differential exposure to environmental conditions, pollutants, and toxins, as well as disparities in the built environment shaping access to and use of fast food, grocery stores, and recreational facilities.

Lastly, it can be thought that education has a weaker association with the adoption of beneficial health behaviors for Black mothers living in nonmetropolitan areas. This hypothesis is partly supported by research suggesting that education is a significant predictor of health consciousness for whites and Hispanics, but not for Blacks (45). This could arise from lower quality education or result from different social dynamics, social network structures and discourse around health in nonmetropolitan Black communities.

These last two potential explanations—access to healthcare and adoption of beneficial health behaviors—are related to our analysis of neonatal and postneonatal mortality insomuch as timing of infant mortality indicates different underlying patterns of causes of death. Because the nonmetropolitan advantage occurs during the neonatal period for Black mothers with less than a high school education, this could suggest that, contrary to initial expectations, it might be harder for mothers with low educational attainment to access quality obstetric and neonatal healthcare in metropolitan counties. Additionally, in all four census regions, Black mothers are much more likely than white mothers to reside in large central metropolitan counties (Table 4). Higher neonatal mortality for the least educated Black women in these areas might result from detrimental contextual conditions, rather than geographical access to healthcare. Moreover, the very similar probabilities of postneonatal death across educational attainment for nonmetropolitan Black mothers suggest that behaviors and environmental conditions may not vary across socioeconomic status. As noted earlier, these factors, which could include child nutrition, parental supervision, smoking, and use of appropriate indoor and outdoor recreational spaces, may be shaped by the built environment.

Although our analysis highlights that nonmetropolitan residence limits Black mothers' returns to education with respect to infant mortality—particularly in the South—, this paper can only hypothesize about the mechanisms underlying this finding. Additional research is needed to shed light on these processes. This paper only provides a partial picture of infant mortality in the U.S. given the sample restrictions, excluding immigrants, Hispanics, Asians, Pacific Islanders, Indigenous peoples, and other ethnoracial groups. We also acknowledge that the data limit us to considering race through categorical identities, despite the complexity of this concept and the fuzzy boundaries between racial groups, especially for multiracial individuals.

A further limitation to this study is the potential for intracategorical variation, both in terms of metropolitan status and education. While our analysis focuses on the distinction between metropolitan and nonmetropolitan counties, the distribution of mothers across the six NCHS urban-rural codes (see Table 4) suggests differences in the locations within the categories of metropolitan and nonmetropolitan where Black and white mothers reside. However, infant mortality is a rare enough occurrence that the data become too sparse to be able to meaningfully compare educational gradients between more fine-grained geographic areas. We also cannot consider intracounty differences in the types of neighborhoods where Black and white mothers live. Thus, further research is needed to continue to understand the heterogeneity within the categories of metropolitan and nonmetropolitan. There is also potential for intracategorical variation in the four-level scale of educational attainment across races and metropolitan county status. For example, we expect higher proportions of college-educated mothers with additional degrees in metropolitan areas, where most research institutions are located, which could contribute to lower levels of postneonatal mortality for college-educated Black mothers in metropolitan counties. Lastly, we want to reiterate that education is not a perfect proxy for socioeconomic status and does not explain all the life course differences between Black and white mothers. Education is acquired with a range of intentions, challenges and results; it also fails to reflect the continuing effects of past socioeconomic status during childhood and other life course experiences. This is especially salient in the health context, as behaviors and health are shaped through childhood.

This paper contributes to the literature on the ways through which mothers' situation and context impact infants' health and mortality. Beyond confirming the persistence of the well-known racial gap in infant mortality and the negative association with maternal educational attainment, we have documented the smaller returns to education for Black mothers living in nonmetropolitan counties, a pattern that is observed across the periods 1998–2002 and 2011–2015. This finding offers a new axis for research and policy intervention focusing on issues relating to limited returns to education for the nonmetropolitan Black population living in the U.S. South. While metropolitan residence and region cannot account for the overall racial gap, the fact that geographic variations are much more salient for Black mothers than for white mothers suggests broader issues related to availability and quality of healthcare and education as well as persistent social stress and discrimination in Black communities. This points to the importance of looking at infant health in a holistic perspective, beyond individual and household characteristics. Considering the geographic dimensions of these dynamics and their persistence over time helps to understand the systemic and ingrained nature of disparities.

## Data availability statement

The data analyzed in this study is subject to the following licenses/restrictions: the datasets analyzed for this study are restricted and can be requested from NCHS. Public versions of these data, which do not include geographic information, can be downloaded from the National Bureau of Economic Research website. Requests to access these datasets should be directed to https://www.cdc.gov/nchs/nvss/nvss-restricted-data.htm.

## Author contributions

MCG wrote the first draft of the manuscript. KDM completed the statistical analysis. MCG and KDM contributed to the conception, manuscript writing and revision, and read and approved the submitted version.

## Funding

This publication was supported by the Princeton University Library Open Access Fund.

## Conflict of interest

The authors declare that the research was conducted in the absence of any commercial or financial relationships that could be construed as a potential conflict of interest.

## Publisher's note

All claims expressed in this article are solely those of the authors and do not necessarily represent those of their affiliated organizations, or those of the publisher, the editors and the reviewers. Any product that may be evaluated in this article, or claim that may be made by its manufacturer, is not guaranteed or endorsed by the publisher.

## Footnotes

1 Analysis was also conducted on those born to non-Hispanic Asian (including Pacific Islanders) or Hispanic (regardless of race) mothers. Results are available upon request. Although we have looked at four ethnoracial groups (non-Hispanic white, non-Hispanic Black, non-Hispanic Asian, and Hispanic), we chose to focus this paper on infants born to white and Black mothers, acknowledging the continued importance of Black-white disparities in the U.S. context. Moreover, large proportions of Hispanic and Asian mothers are first-generation immigrants who were dropped from this analysis, and a considerable number of U.S.-born individuals from these communities are second- or third-generation immigrants. In light of the health selection of immigrants (27) and evidence suggesting partial intergenerational transmission of the healthy immigrant advantage (11–14), the educational gradient in infant mortality for Hispanic and Asian mothers can be assumed to derive from distinct mechanisms than for white and Black mothers, who largely belong to communities that have been in the United States for multiple generations.

2 The code for this paper's analysis was adapted from Green and Hamilton (6), who made replication material available on the website of *Demographic Research*.
