# Supplementary material for: Geographic heterogeneity in Black-white infant mortality disparities
Source: Front Public Health. 2022 Nov 3;10:995585. doi: 10.3389/fpubh.2022.995585 (PMC9669983; doi:10.3389/fpubh.2022.995585)
Supplement: Supplementary file 1 [file Data_Sheet_1.PDF]

Supplementary Material

1 SUPPLEMENTARY TABLES AND FIGURES

1.1 Tables

Table S1. Logistic regression models of infant mortality stratified by race, subset of states, 2011-2015

	Whites		Blacks	
	**Model 1**	**Model 2**	**Model 1**	**Model 2**
**Maternal demographic characteristics**				
Nonmetropolitan	0.122	0.117	0.357	0.387
	(-0.048, 0.292)	(-0.054, 0.287)	(-0.349, 1.063)	(-0.319, 1.094)
Maternal age	-0.137***	-0.106***	-0.049***	-0.004
	(-0.153, -0.121)	(-0.122, -0.089)	(-0.070, -0.027)	(-0.027, 0.019)
Maternal age^2	0.002***	0.002***	0.001***	0.000**
	(0.002, 0.003)	(0.002, 0.002)	(0.001, 0.002)	(0.000, 0.001)
Mother married		-0.317***		-0.097***
		(-0.342, -0.292)		(-0.138, -0.057)
**Maternal education**				
High school	-0.415***	-0.328***	-0.048	-0.048
	(-0.530, -0.299)	(-0.443, -0.213)	(-0.182, 0.087)	(-0.183, 0.087)
Some college	-0.722***	-0.559***	-0.422***	-0.421***
	(-0.838, -0.607)	(-0.676, -0.443)	(-0.563, -0.281)	(-0.563, -0.280)
College +	-1.337***	-1.021***	-0.666***	-0.657***
	(-1.452, -1.221)	(-1.139, -0.904)	(-0.849, -0.483)	(-0.842, -0.473)
**Nonmetro * Educ**				
Nonmetro * HS	0.04	0.038	-0.343	-0.354
	(-0.173, 0.253)	(-0.176, 0.251)	(-1.239, 0.554)	(-1.251, 0.542)
Nonmetro * Some college	0.059	0.049	-0.592	-0.597
	(-0.163, 0.282)	(-0.174, 0.272)	(-1.587, 0.403)	(-1.593, 0.398)
Nonmetro * College +	0.438***	0.413***	0.07	0.059
	(0.196, 0.681)	(0.171, 0.656)	(-1.151, 1.290)	(-1.162, 1.279)
**Child characteristics**				
Child male		0.210***		0.179***
		(0.188, 0.231)		(0.150, 0.209)
1 prior birth		-0.047***		-0.243***
		(-0.074, -0.020)		(-0.281, -0.205)
2 prior births		0.106***		-0.238***
		(0.073, 0.139)		(-0.284, -0.193)
3 or more prior births		0.261***		-0.140***
		(0.223, 0.299)		(-0.189, -0.092)
1st trimester prenatal care		-0.296***		-0.158***
		(-0.321, -0.272)		(-0.188, -0.127)
**Region of birth**				
Midwest	0.154***	0.130**	0.209***	0.225***
	(0.041, 0.267)	(0.017, 0.244)	(0.082, 0.335)	(0.098, 0.352)
South	0.130**	0.117**	0.134**	0.144**
	(0.021, 0.238)	(0.008, 0.226)	(0.014, 0.253)	(0.025, 0.264)
West	0.025	0.027	-0.038	-0.019
	(-0.103, 0.154)	(-0.102, 0.156)	(-0.211, 0.134)	(-0.192, 0.153)

Table S1. (continued)

**Year of birth**				
2012		-0.011		0.012
		(-0.045, 0.024)		(-0.036, 0.060)
2013		-0.014		-0.028
		(-0.048, 0.021)		(-0.075, 0.020)
2014		0.015		0.012
		(-0.019, 0.049)		(-0.034, 0.059)
2015		0.012		0.039*
		(-0.022, 0.045)		(-0.007, 0.085)
**Region * Nonmetro**				
Midwest * Nonmetro	-0.152	-0.116	-0.359	-0.378
	(-0.348, 0.045)	(-0.313, 0.081)	(-1.152, 0.434)	(-1.171, 0.415)
South * Nonmetro	-0.025	0.009	-0.468	-0.485
	(-0.218, 0.167)	(-0.183, 0.202)	(-1.184, 0.249)	(-1.201, 0.232)
West * Nonmetro	-0.085	-0.065	-0.164	-0.189
	(-0.334, 0.163)	(-0.313, 0.184)	(-1.884, 1.556)	(-1.909, 1.531)
**Region * Educ**				
Midwest * HS	0.078	0.089	-0.059	-0.063
	(-0.061, 0.216)	(-0.050, 0.227)	(-0.216, 0.098)	(-0.220, 0.094)
Midwest * Some college	0.096	0.117*	0.171**	0.169**
	(-0.042, 0.233)	(-0.020, 0.255)	(0.009, 0.334)	(0.007, 0.332)
Midwest * College +	0.197***	0.215***	0.01	0.016
	(0.060, 0.335)	(0.077, 0.353)	(-0.215, 0.234)	(-0.208, 0.240)
South * HS	0.148**	0.160**	-0.063	-0.064
	(0.016, 0.280)	(0.027, 0.292)	(-0.210, 0.084)	(-0.211, 0.084)
South * Some college	0.098	0.114*	0.118	0.12
	(-0.035, 0.231)	(-0.019, 0.247)	(-0.036, 0.272)	(-0.034, 0.273)
South * College +	0.171**	0.177***	0.076	0.082
	(0.038, 0.305)	(0.043, 0.311)	(-0.123, 0.274)	(-0.116, 0.280)
West * HS	0.051	0.081	-0.073	-0.071
	(-0.104, 0.206)	(-0.074, 0.236)	(-0.283, 0.137)	(-0.281, 0.139)
West * Some college	0.093	0.117	0.134	0.143
	(-0.060, 0.246)	(-0.036, 0.270)	(-0.080, 0.348)	(-0.071, 0.357)
West * College +	0.264***	0.259***	-0.133	-0.122
	(0.111, 0.418)	(0.105, 0.412)	(-0.426, 0.160)	(-0.415, 0.171)
**Region * Nonmetro * Educ**				
Midwest * Nonmetro * HS	0.076	0.058	0.181	0.197
	(-0.170, 0.322)	(-0.188, 0.303)	(-0.821, 1.184)	(-0.806, 1.200)
Midwest * Nonmetro * SC	-0.017	-0.037	0.393	0.408
	(-0.270, 0.237)	(-0.291, 0.217)	(-0.704, 1.489)	(-0.689, 1.505)
Midwest * Nonmetro * C+	-0.273*	-0.305**	-0.492	-0.485
	(-0.553, 0.007)	(-0.585, -0.026)	(-2.043, 1.058)	(-2.036, 1.065)
South * Nonmetro * HS	-0.034	-0.049	0.43	0.44
	(-0.273, 0.206)	(-0.289, 0.191)	(-0.478, 1.338)	(-0.468, 1.348)
South * Nonmetro * SC	0.007	-0.012	0.829	0.826
	(-0.243, 0.256)	(-0.262, 0.238)	(-0.177, 1.835)	(-0.180, 1.833)
South * Nonmetro * C+	-0.363**	-0.380***	0.254	0.251
	(-0.644, -0.081)	(-0.661, -0.098)	(-0.988, 1.497)	(-0.992, 1.493)

Table S1. (continued)

West * Nonmetro * HS	-0.003	-0.035	-0.728	-0.71
	(-0.308, 0.301)	(-0.339, 0.269)	(-2.863, 1.406)	(-2.845, 1.425)
West * Nonmetro * SC	0.056	0.026	0.285	0.288
	(-0.251, 0.363)	(-0.281, 0.333)	(-1.700, 2.270)	(-1.697, 2.273)
West * Nonmetro * C+	-0.254	-0.290*	-	-
	(-0.596, 0.087)	(-0.631, 0.051)		
**Constant**	-2.967***	-3.531***	-3.958***	-4.702***
	(-3.521, -2.413)	(-4.044, -3.018)	(-4.152, -3.765)	(-4.978, -4.426)
**Observations**	8,394,020	8,394,020	1,949,362	1,949,362

Robust ci in parentheses
*** $p < 0.01$, ** $p < 0.05$, * $p < 0.1$

Table S2. Logistic regression models of neonatal mortality stratified by race, 2011-2015

	Whites		Blacks	
	**Model 1**	**Model 2**	**Model 1**	**Model 2**
**Maternal demographic characteristics**				
Nonmetropolitan	0.084***	0.088***	-0.098*	-0.087**
	(0.031, 0.137)	(0.023, 0.154)	(-0.209, 0.012)	(-0.156, -0.017)
Maternal age	-0.126***	-0.060***	-0.047***	0.068***
	(-0.153, -0.098)	(-0.087, -0.032)	(-0.072, -0.023)	(0.030, 0.106)
Maternal age^2	0.002***	0.001***	0.001***	0
	(0.002, 0.003)	(0.001, 0.002)	(0.001, 0.002)	(-0.001, 0.000)
Mother married		-0.221***		-0.038
		(-0.298, -0.143)		(-0.093, 0.017)
**Maternal education**				
High school	-0.225***	-0.185***	0.035***	-0.026
	(-0.289, -0.161)	(-0.247, -0.124)	(0.012, 0.059)	(-0.059, 0.008)
Some college	-0.504***	-0.436***	-0.097***	-0.210***
	(-0.554, -0.455)	(-0.499, -0.374)	(-0.139, -0.055)	(-0.281, -0.140)
College +	-0.943***	-0.816***	-0.350***	-0.581***
	(-1.051, -0.836)	(-0.917, -0.716)	(-0.377, -0.323)	(-0.647, -0.515)
**Nonmetro * Educ**				
Nonmetro * HS	0.027	0.026	0.087***	0.086***
	(-0.028, 0.082)	(-0.033, 0.085)	(0.035, 0.140)	(0.033, 0.138)
Nonmetro * Some college	0.041***	0.035***	0.177***	0.177***
	(0.020, 0.061)	(0.022, 0.048)	(0.162, 0.191)	(0.156, 0.198)
Nonmetro * College +	0.198***	0.179***	0.172***	0.185***
	(0.064, 0.331)	(0.053, 0.306)	(0.076, 0.269)	(0.066, 0.304)
**Child characteristics**				
Child male		0.160***		0.175***
		(0.133, 0.186)		(0.132, 0.219)
1 prior birth		-0.293***		-0.520***
		(-0.313, -0.273)		(-0.590, -0.451)
2 prior births		-0.208***		-0.603***
		(-0.254, -0.161)		(-0.721, -0.484)
3 or more prior births		-0.059		-0.622***
		(-0.160, 0.042)		(-0.738, -0.507)
1st trimester prenatal care		-0.254***		-0.097***
		(-0.322, -0.186)		(-0.131, -0.064)
**Region of birth**				
Midwest		0.205***		0.281***
		(0.191, 0.220)		(0.267, 0.296)
South		0.132***		0.165***
		(0.122, 0.142)		(0.160, 0.171)
West		0.120***		-0.067***
		(0.113, 0.126)		(-0.074, -0.060)

Table S2. (continued)

**Year of birth**				
2012		-0.008		-0.016
		(-0.054, 0.038)		(-0.047, 0.015)
2013		0.02		-0.048***
		(-0.021, 0.060)		(-0.072, -0.024)
2014		0.025**		-0.027**
		(0.006, 0.044)		(-0.051, -0.003)
2015		0.004		-0.031**
		(-0.040, 0.048)		(-0.059, -0.002)
**Constant**	-3.979***	-4.878***	-4.745***	-6.340***
	(-4.442, -3.516)	(-5.339, -4.418)	(-5.088, -4.402)	(-6.899, -5.781)
**Observations**	8,394,020	8,394,020	1,949,362	1,949,362

Robust ci in parentheses
*** $p < 0.01$, ** $p < 0.05$, * $p < 0.1$

Table S3. Logistic regression models of postneonatal mortality stratified by race, 2011-2015

	Whites		Blacks	
	**Model 1**	**Model 2**	**Model 1**	**Model 2**
**Maternal demographic characteristics**				
Nonmetropolitan	0.033	0.048	-0.088	-0.102*
	(-0.074, 0.141)	(-0.030, 0.125)	(-0.206, 0.030)	(-0.215, 0.010)
Maternal age	-0.132***	-0.165***	-0.032	-0.126***
	(-0.190, -0.074)	(-0.221, -0.109)	(-0.071, 0.008)	(-0.164, -0.087)
Maternal age^2	0.002***	0.002***	0.001	0.002***
	(0.001, 0.003)	(0.001, 0.003)	(-0.000, 0.001)	(0.001, 0.003)
Mother married		-0.453***		-0.219***
		(-0.519, -0.387)		(-0.331, -0.108)
**Maternal education**				
High school	-0.436***	-0.242***	-0.286***	-0.183***
	(-0.513, -0.359)	(-0.319, -0.165)	(-0.305, -0.268)	(-0.187, -0.179)
Some college	-0.790***	-0.446***	-0.585***	-0.381***
	(-0.834, -0.746)	(-0.488, -0.405)	(-0.607, -0.563)	(-0.421, -0.341)
College +	-1.489***	-0.858***	-1.115***	-0.660***
	(-1.586, -1.392)	(-0.971, -0.745)	(-1.266, -0.964)	(-0.734, -0.587)
**Nonmetro * Educ**				
Nonmetro * HS	0.096**	0.05	0.022	0.016
	(0.006, 0.185)	(-0.017, 0.118)	(-0.064, 0.109)	(-0.067, 0.099)
Nonmetro * Some college	0.078***	0.02	0.225*	0.205
	(0.036, 0.120)	(-0.028, 0.068)	(-0.043, 0.493)	(-0.047, 0.457)
Nonmetro * College +	0.076	-0.017	0.632***	0.561***
	(-0.104, 0.257)	(-0.162, 0.128)	(0.475, 0.789)	(0.406, 0.717)
**Child characteristics**				
Child male		0.283***		0.185***
		(0.249, 0.318)		(0.153, 0.218)
1 prior birth		0.345***		0.264***
		(0.286, 0.404)		(0.186, 0.342)
2 prior births		0.596***		0.411***
		(0.535, 0.656)		(0.348, 0.473)
3 or more prior births		0.783***		0.687***
		(0.699, 0.868)		(0.599, 0.774)
1st trimester prenatal care		-0.348***		-0.238***
		(-0.371, -0.325)		(-0.260, -0.217)
**Region of birth**				
Midwest		0.218***		0.204***
		(0.214, 0.221)		(0.197, 0.211)
South		0.383***		0.169***
		(0.381, 0.386)		(0.165, 0.173)
West		0.152***		0.062***
		(0.147, 0.156)		(0.055, 0.069)

Table S3. (continued)

**Year of birth**				
2012		-0.013		0.058***
		(-0.069, 0.043)		(0.019, 0.098)
2013		-0.063		0.009
		(-0.138, 0.013)		(-0.057, 0.076)
2014		0.002		0.079***
		(-0.030, 0.034)		(0.022, 0.136)
2015		0.023		0.153***
		(-0.020, 0.066)		(0.131, 0.176)
**Constant**	-3.567***	-3.448***	-4.755***	-3.830***
	(-4.342, -2.792)	(-4.298, -2.598)	(-5.340, -4.170)	(-4.352, -3.307)
**Observations**	8,373,608	8,373,608	1,937,934	1,937,934

Robust ci in parentheses
*** $p < 0.01$, ** $p < 0.05$, * $p < 0.1$

Table S4. Logistic regression models of infant mortality stratified by race, 1998-2002

	**Whites**		**Blacks**	
	**Model 1**	**Model 2**	**Model 1**	**Model 2**
**Maternal demographic characteristics**				
Nonmetropolitan	0.040	0.044	-0.025	-0.014
	(-0.047, 0.126)	(-0.048, 0.136)	(-0.118, 0.069)	(-0.039, 0.011)
Maternal age	-0.188***	-0.150***	-0.007	0.036**
	(-0.199, -0.176)	(-0.164, -0.135)	(-0.027, 0.014)	(0.008, 0.065)
Maternal age^2	0.003***	0.003***	0.000*	-0.000
	(0.003, 0.003)	(0.002, 0.003)	(-0.000, 0.001)	(-0.001, 0.000)
Mother married		-0.292***		-0.130***
		(-0.348, -0.236)		(-0.175, -0.085)
**Maternal education**				
12 years	-0.390***	-0.278***	-0.156***	-0.139***
	(-0.413, -0.367)	(-0.313, -0.243)	(-0.185, -0.128)	(-0.149, -0.129)
13-15 years	-0.686***	-0.518***	-0.349***	-0.315***
	(-0.728, -0.643)	(-0.568, -0.468)	(-0.382, -0.315)	(-0.340, -0.289)
16+ years	-1.004***	-0.766***	-0.563***	-0.513***
	(-1.055, -0.953)	(-0.817, -0.715)	(-0.662, -0.463)	(-0.566, -0.461)
**Nonmetro * Educ**				
Nonmetro * 12 years	0.062**	0.034	0.002	-0.015
	(0.001, 0.124)	(-0.024, 0.091)	(-0.063, 0.068)	(-0.066, 0.036)
Nonmetro * 13-15 years	0.116***	0.077**	0.164***	0.136***
	(0.052, 0.181)	(0.009, 0.144)	(0.111, 0.217)	(0.071, 0.201)
Nonmetro * 16+ years	0.176***	0.125**	0.246***	0.212***
	(0.047, 0.305)	(0.008, 0.242)	(0.165, 0.327)	(0.156, 0.267)
**Child characteristics**				
Child male		0.223***		0.200***
		(0.202, 0.244)		(0.186, 0.214)
1 prior birth		-0.046*		-0.265***
		(-0.097, 0.005)		(-0.300, -0.230)
2 prior births		0.081***		-0.219***
		(0.021, 0.141)		(-0.252, -0.186)
3 or more prior births		0.272***		-0.092***
		(0.202, 0.342)		(-0.128, -0.057)
1st trimester prenatal care		-0.250***		-0.117***
		(-0.292, -0.207)		(-0.155, -0.079)
**Region of birth**				
Midwest		0.238***		0.281***
		(0.220, 0.257)		(0.279, 0.283)
South		0.251***		0.180***
		(0.232, 0.269)		(0.174, 0.186)
West		0.128***		0.011***
		(0.120, 0.137)		(0.006, 0.017)

Table S4. (continued)

**Year of birth**				
1999		-0.036**		0.016
		(-0.065, -0.008)		(-0.004, 0.037)
2000		-0.011		0.010
		(-0.038, 0.017)		(-0.024, 0.044)
2001		-0.025		-0.023***
		(-0.067, 0.017)		(-0.029, -0.016)
2002		-0.016		0.001
		(-0.080, 0.048)		(-0.011, 0.013)
**Constant**	-2.239***	-2.819***	-4.316***	-5.009***
	(-2.377, -2.101)	(-3.027, -2.612)	(-4.639, -3.992)	(-5.368, -4.649)
**Observations**	10,093,743	10,093,743	2,209,892	2,209,892

Robust ci in parentheses

*** $p < 0.01$, ** $p < 0.05$, * $p < 0.1$

Table S5. Logistic regression models of infant mortality stratified by race, subset of states, 2011-2015

	Whites		Blacks	
	**Model 1**	**Model 2**	**Model 1**	**Model 2**
**Maternal demographic characteristics**				
Nonmetropolitan	0.064	0.074	-0.063**	-0.058***
	(-0.033, 0.161)	(-0.021, 0.169)	(-0.118, -0.007)	(-0.099, -0.017)
Maternal age	-0.144***	-0.114***	-0.048***	-0.004
	(-0.186, -0.101)	(-0.149, -0.078)	(-0.069, -0.028)	(-0.026, 0.019)
Maternal age^2	0.003***	0.002***	0.001***	0.000***
	(0.002, 0.003)	(0.001, 0.003)	(0.001, 0.001)	(0.000, 0.001)
Mother married		-0.302***		-0.110***
		(-0.362, -0.241)		(-0.173, -0.047)
**Maternal education**				
High school	-0.317***	-0.217***	-0.095***	-0.093***
	(-0.380, -0.255)	(-0.285, -0.148)	(-0.119, -0.072)	(-0.109, -0.077)
Some college	-0.633***	-0.458***	-0.291***	-0.282***
	(-0.642, -0.625)	(-0.494, -0.422)	(-0.329, -0.254)	(-0.343, -0.222)
College +	-1.142***	-0.824***	-0.617***	-0.585***
	(-1.217, -1.068)	(-0.909, -0.739)	(-0.678, -0.556)	(-0.617, -0.552)
**Nonmetro * Educ**				
Nonmetro * HS	0.048	0.03	0.003	0
	(-0.043, 0.138)	(-0.055, 0.114)	(-0.062, 0.068)	(-0.065, 0.065)
Nonmetro * Some college	0.050***	0.024**	0.152*	0.144*
	(0.018, 0.082)	(0.000, 0.047)	(-0.010, 0.313)	(-0.017, 0.304)
Nonmetro * College +	0.124**	0.079*	0.292***	0.270***
	(0.027, 0.222)	(-0.013, 0.170)	(0.164, 0.420)	(0.118, 0.421)
**Child characteristics**				
Child male		0.208***		0.186***
		(0.178, 0.238)		(0.141, 0.232)
1 prior birth		-0.039**		-0.229***
		(-0.073, -0.006)		(-0.291, -0.167)
2 prior births		0.122***		-0.230***
		(0.067, 0.178)		(-0.312, -0.148)
3 or more prior births		0.261***		-0.131***
		(0.158, 0.363)		(-0.226, -0.036)
1st trimester prenatal care		-0.298***		-0.172***
		(-0.328, -0.268)		(-0.203, -0.141)
**Region of birth**				
Midwest		0.207***		0.233***
		(0.199, 0.215)		(0.222, 0.243)
South		0.225***		0.146***
		(0.222, 0.228)		(0.141, 0.151)
West		0.123***		-0.032***
		(0.119, 0.127)		(-0.036, -0.027)

Table S5. (continued)

**Year of birth**				
2012		-0.01		0.008
		(-0.052, 0.031)		(-0.006, 0.023)
2013		-0.016		-0.033*
		(-0.061, 0.029)		(-0.068, 0.003)
2014		0.002		0.011
		(-0.011, 0.014)		(-0.008, 0.029)
2015		0.003		0.025**
		(-0.033, 0.039)		(0.002, 0.047)
**Constant**	-2.914***	-3.426***	-3.982***	-4.688***
	(-3.530, -2.298)	(-3.995, -2.858)	(-4.207, -3.757)	(-5.009, -4.367)
**Observations**	7,520,860	7,520,860	1,773,378	1,773,378

Robust ci in parentheses

*** $p < 0.01$, ** $p < 0.05$, * $p < 0.1$

Table S6. Logistic regression models of infant mortality stratified by race, subset of states, 1998-2002

	Whites		Blacks	
	**Model 1**	**Model 2**	**Model 1**	**Model 2**
**Maternal demographic characteristics**				
Nonmetropolitan	0.060**	0.067**	0.027	0.034**
	(0.011, 0.108)	(0.014, 0.120)	(-0.022, 0.075)	(0.002, 0.067)
Maternal age	-0.194***	-0.156***	0.009***	0.054***
	(-0.205, -0.183)	(-0.171, -0.140)	(0.004, 0.015)	(0.047, 0.061)
Maternal age^2	0.003***	0.003***	0	-0.001***
	(0.003, 0.004)	(0.002, 0.003)	(-0.000, 0.000)	(-0.001, -0.001)
Mother married		-0.289***		-0.138***
		(-0.332, -0.247)		(-0.156, -0.121)
**Maternal education**				
12 years	-0.386***	-0.274***	-0.129***	-0.118***
	(-0.413, -0.359)	(-0.314, -0.234)	(-0.155, -0.103)	(-0.133, -0.102)
13-15 years	-0.691***	-0.524***	-0.344***	-0.317***
	(-0.731, -0.652)	(-0.571, -0.477)	(-0.412, -0.276)	(-0.361, -0.273)
16+ years	-1.012***	-0.775***	-0.527***	-0.484***
	(-1.072, -0.951)	(-0.834, -0.717)	(-0.612, -0.441)	(-0.518, -0.449)
**Nonmetro * Educ**				
Nonmetro * 12 years	0.042	0.014	-0.043*	-0.054***
	(-0.023, 0.107)	(-0.043, 0.072)	(-0.088, 0.002)	(-0.093, -0.014)
Nonmetro * 13-15 years	0.099***	0.060***	0.174***	0.156***
	(0.056, 0.142)	(0.020, 0.100)	(0.151, 0.198)	(0.126, 0.187)
Nonmetro * 16+ years	0.198**	0.150**	0.236***	0.212***
	(0.038, 0.358)	(0.008, 0.292)	(0.205, 0.268)	(0.174, 0.250)
**Child characteristics**				
Child male		0.220***		0.210***
		(0.204, 0.237)		(0.190, 0.231)
1 prior birth		-0.039		-0.259***
		(-0.102, 0.024)		(-0.309, -0.208)
2 prior births		0.090**		-0.214***
		(0.014, 0.166)		(-0.277, -0.151)
3 or more prior births		0.277***		-0.095***
		(0.211, 0.342)		(-0.154, -0.035)
1st trimester prenatal care		-0.267***		-0.134***
		(-0.320, -0.213)		(-0.218, -0.051)
**Region of birth**				
Midwest		0.226***		0.199***
		(0.206, 0.246)		(0.194, 0.204)
South		0.212***		0.107***
		(0.197, 0.227)		(0.098, 0.117)
West		0.111***		0.010**
		(0.098, 0.125)		(0.002, 0.017)

Table S6. (continued)

**Year of birth**				
1999		-0.027***		0.019
		(-0.047, -0.007)		(-0.012, 0.050)
2000		-0.003		-0.007
		(-0.018, 0.012)		(-0.056, 0.042)
2001		-0.017		-0.005
		(-0.064, 0.029)		(-0.052, 0.041)
2002		0.003		0.009
		(-0.040, 0.045)		(-0.027, 0.044)
**Constant**	-2.175***	-2.721***	-4.552***	-5.195***
	(-2.304, -2.046)	(-2.886, -2.556)	(-4.639, -4.466)	(-5.305, -5.085)
**Observations**	6,879,306	6,879,306	1,478,063	1,478,063

Robust ci in parentheses
*** $p < 0.01$, ** $p < 0.05$, * $p < 0.1$
